# Baseline patient reported outcomes are more consistent predictors of long-term functional disability than laboratory, imaging or joint count data in patients with early inflammatory arthritis: A systematic review

**DOI:** 10.1016/j.semarthrit.2018.03.004

**Published:** 2018-12

**Authors:** James M. Gwinnutt, Charlotte A. Sharp, Deborah P.M. Symmons, Mark Lunt, Suzanne M.M. Verstappen

**Affiliations:** a Arthritis Research UK Centre for Epidemiology, Centre for Musculoskeletal Research, Faculty of Biology, Medicine and Health, University of Manchester, Manchester, UK; b National Institute for Health Research Collaboration for Leadership in Applied Health Research and Care (NIHR CLAHRC) Greater Manchester, Greater Manchester, Alliance Manchester Business School, The University of Manchester, UK; c NIHR Manchester Biomedical Research Centre, Manchester University Hospitals NHS Foundation Trust, Manchester Academic Health Science Centre, UK

**Keywords:** Early rheumatoid arthritis, Long-term outcome, Functional disability, Systematic review

## Abstract

**Objective:**

To assess baseline predictors of long-term functional disability in patients with inflammatory arthritis (IA).

**Methods:**

We conducted a systematic review of the literature from 1990 to 2017 using MEDLINE and EMBASE. Studies were included if (i) they were prospective observational studies, (ii) all patients had IA with symptom duration ≤2 years at baseline, (iii) follow-up was at least 5 years, and (iv) baseline predictors of HAQ score at long-term follow-up (i.e., ≥5 years following baseline) were assessed. Information on the included studies and estimates of the association between baseline variables and long-term HAQ scores were extracted from the full manuscripts.

**Results:**

Of 1037 abstracts identified by the search strategy, 37 met the inclusion/exclusion criteria and were included in the review. Older age at baseline and female gender were reported to be associated with higher long-term HAQ scores in the majority of studies assessing these relationships, as were higher baseline HAQ and greater pain scores (total patients included in analyses reporting significant associations/total number of patients analysed: age 9.8k/10.7k (91.6%); gender 9.9k/11.3k (87.4%); HAQ 4.0k/4.0k (99.0%); pain 2.8k/2.9k (93.6%)). Tender joint count, erythrocyte sedimentation rate (ESR) and DAS28 were also reported to predict long-term HAQ score; other disease activity measures were less consistent (tender joints 2.1k/2.5k (84.5%); erythrocyte sedimentation rate 1.6k/2.2k (72.3%); DAS28 888/1.1k (79.2%); swollen joints 684/2.6k (26.6%); C-reactive protein 279/510 (54.7%)). Rheumatoid factor (RF) and erosions were not useful predictors (RF 546/4.6k (11.9%); erosions 191/2.7k (7.0%)), whereas the results for anti-citrullinated protein antibody positivity were equivocal (ACPA 2.0k/3.8k (52.9%)).

**Conclusions:**

Baseline age, gender, HAQ and pain scores are associated with long-term disability and knowledge of these may aid the assessment of prognosis.

## Introduction

Inflammatory arthritis (IA), and its subset rheumatoid arthritis (RA), are chronic conditions characterised by synovial joint inflammation [1]. Negative outcomes associated with these conditions include premature mortality [2], [3], joint destruction [4], [5], and functional disability [6], [7], [8]. The term functional disability refers to the difficulties patients with IA have in performing everyday tasks. Preventing or minimising functional disability is a key goal in IA management.

In the past, functional disability was assessed using the Steinbrocker Functional Class system, in which the physician scored the patient from class 1 (indicating little or no disability), to class 4 (indicating patients were bed-ridden or confined to a wheel chair) [9]. Whilst this system was quick and reflected clinicians’ judgement, only having four levels of disability meant the measure was insensitive to change [10]. Later the Health Assessment Questionnaire – Disability Index (HAQ) was developed [11]. The HAQ comprises 20 questions in eight subsections assessing different aspects of everyday life, yielding a score of 0–3, with 0 indicating no disability and 3 representing substantial levels of disability. The HAQ has become the gold standard for measuring disability in patients with IA and has been shown to be a valid measure of disability [12], [13]. A minimum clinically important difference was estimated to be between 0.20 and 0.22 [14], although later estimates have put the value as low as 0.09 within an observational cohort setting [15].

Longitudinally, functional disability measured using the HAQ has been shown to follow a J-shaped trajectory, with initial improvements in disability one to two years following symptom onset, followed by increasing HAQ scores over the subsequent 5–10 years [6]. Being able to predict which patients are likely to develop major problems in performing daily tasks is useful for patients and clinicians. Clinicians can target patients susceptible to high levels of long-term disability to receive additional interventions alongside their pharmacological therapy. Patients too may be able to modify their lifestyle to reduce future disability. A systematic review of predictors of HAQ score in patients with RA was published in 2003 [16]. A further literature review was published in 2010 including studies with patients with a range of disease durations at baseline (<1 to 12 years) and follow-up lengths (1–15 years) [17]. However, the latter was not a systematic review and since 2003 a number of additional manuscripts investigating predictors of functional disability have been published. Furthermore, due to the J-shaped trajectory of functional disability, baseline predictors of short term (i.e., between 0 and 5 years) HAQ score may not be the same as predictors of long-term (i.e., ≥5 years) HAQ. Therefore, it is important to consider predictors of long-term functional disability separately from predictors of short-term functional disability, as measured by the HAQ.

The aim of this systematic review was to critically evaluate the available literature on baseline predictors of long-term (i.e., ≥5 years) functional disability in patients with early IA.

## Methods

To address these aims, we performed a systematic review using the MEDLINE and EMBASE databases, including studies published between 01/01/1990 and 05/10/2017. The inclusion criteria were (i) all patients had IA (≥2 swollen joints lasting for ≥4 weeks), RA (defined as meeting any of the published criteria sets [18], [19], [20]), or undifferentiated arthritis; (ii) all patients had less than or equal to two years symptom duration at baseline; (iii) analysis had to assess baseline predictors of long-term functional disability measured using the HAQ at ≥5 years following baseline; (iv) studies had to be observational; (v) studies published in English (or a translation available). Exclusion criteria were (i) randomised controlled trials, clinical trials, cross-sectional studies or case-series; (ii) studies including children; (iii) studies including non-human animals; (iv) conference abstracts. The study was designed and reported according to PRISMA guidelines [21].

A search strategy was devised which included both text words and MESH terms (Supplementary file 1). This search strategy yielded 1037 titles and abstracts, 532 from MEDLINE and 505 from EMBASE. Of these 263 were identified as duplicates by reference managing software (Endnote) and were removed.

**Fig. 1:**
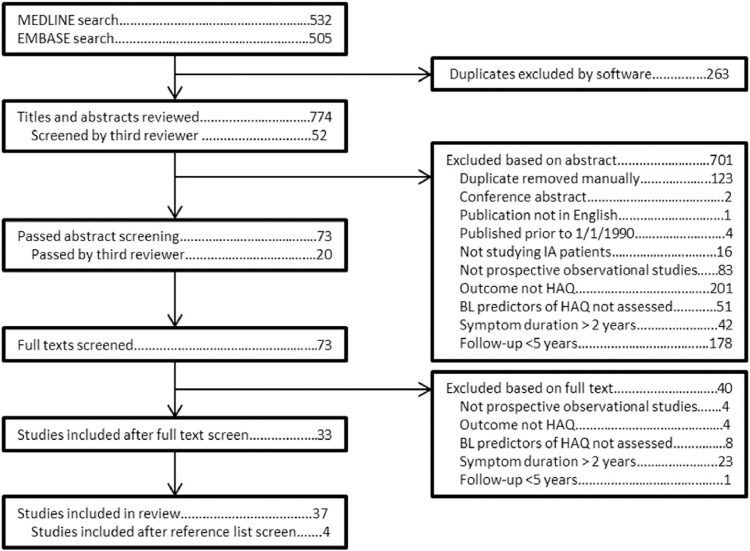
A flow-diagram of the screening strategy. BL = baseline, HAQ = Health Assessment Questionnaire, IA = inflammatory arthritis, N = number.

Each of the remaining titles and abstracts was independently screened based on the inclusion and exclusion criteria by two reviewers using a standardized form (JG and CS). In case of any discrepancies in agreement between the two reviewers (*n* = 53) a third reviewer was consulted (SV). Of 774 titles and abstracts screened, 73 met the inclusion criteria and the full manuscript was read by the same reviewers. Of these, 33 papers were included in the review. The reference lists of these manuscripts were screened. Four additional studies were added to the review, meaning a total of 37 studies were included (Fig. 1).

### Quality assessment

Two reviewers assessed the conduct and reporting of each study using a system adapted from Pasma et al. [22]. Details on the methods and results of the quality assessment can be found in Supplementary file 2.

### Data abstraction

A data abstraction form was created to extract and summarise information from each included study (see data abstraction form in Supplementary file 3), including: number of patients in each study, the length of follow-up, age, gender, baseline and follow-up HAQ scores and information on analyses carried out assessing the association between baseline predictors and follow-up HAQ score. The predictors of long-term HAQ score were grouped into five categories and presented in tables: demographics, patient reported outcomes, disease activity, autoantibody status and miscellaneous. Each of these tables (i.e., other than Table 1, Table 2) displays results from studies that performed multivariable analyses first, followed by studies that only performed univariable analyses. Within these subsections the studies were sorted by sample size. The statistical method of each analysis is reported, followed by effect sizes with 95% confidence intervals.

**Table 1:** Descriptive statistics of included studies

Study	Country	*N*	Age (years), mean (SD) unless otherwise stated	Women (%)	Follow-up (years)	HAQ – baseline, mean (SD) unless otherwise stated	HAQ – final follow-up, mean (SD) unless otherwise stated
Ahlmen [4]	SW	549	Women 54 (16)	63	5	Women 1.1 (0.6)	Women 0.7 (0.7)
			Men 61 (13)			Men 0.8 (0.6)	Men 0.5 (0.6)
Ajeganova [26]	SW	1596	55.6 (14.6)	68	15	1.0 (0.6)	0.6 (0.6)
Andersson [43]	SW	1430	Immigrants (I) 55 (13)	I 76	15	I 1.2 (0.7)	I 0.7
			Non-immigrants (non-I) 55 (14)	non-I 70		Non-I 1.0 (0.6)	Non-I 0.6 (no SDs reported)
Bansback [44]	UK	985	Median 55	66	5	Median 1.0	–
Benton [27]	NZ	42	Median (range) 48.5 (27–75)	62	6	Median (range) 0.6 (0–1.8)	Median (range) 0.3 (0–1.6)
Bjork [49]	SW	189	Women 53 (15)	69	5	Women 0.9 (0.6)	–
			Men 58 (13)			Men 0.8 (0.5)	
Burr [50]	UK	463	Median (IQR) 55.4 (45.8, 65.4)	66	5	Median (IQR) 0.9 (0.4, 1.5)	Median (IQR) 1.1 (0.4, 1.8)
Camacho [24]	UK	3666	<55 (%)/55–74 (%)/≥75 (%)	66	15	Median (IQR)	–
			Women 53.6/37.6/8.8			Women 0.9 (0.4, 1.6)	
			Men 41.3/46.0/12.7			Men 0.6 (0.1, 1.3)	
Camacho [29]	UK	1872	Median (IQR)Parous 54.3 (44.6, 65.2)Nulliparous 54.1 (36.4, 70.0)	100	15	Median (IQR) Parous 0.9 (0.4,1.6)Nulliparous 0.9 (0.4, 1.6)	–
							
Combe [34]	FR	191	50.5 (14.7)	73	5	Median (range) 1.3 (0, 2.8)	Median (range) 0.6 (0, 3.0)
Combe [51]	FR	813	48.1 (12.6)	77	5	1.0 (0.7)	0.5 (0.6)
Contreras-Yanez [25]	MX	107	39.1 (13.3)	89	5	1.5 (0.9, 2.1)	–
Eberhardt [28]	SW	63	52.4 (13.7)	62	5	–	Women 0.9 (0.6)
							Men 0.4 (0.4)
Eberhardt [52]	SW	99	52.1 (12.8)	67	5	–	Medians ranged from 1.0 to 1.4 depending on immunogenetic group
Forslind [46]	SW	92	53 (15)	66	5	Median (IQR) 1.0 (0.6, 1.4)	0.4 (0.0, 1.1)
Genevay [23]	FR	25	50.6 (15.5)	72	avg 8.5	0.8 (0.8)	–
Hallert [35]	SW	251	Women 55 (16)	65	8	Women 0.9 (0.6)	Women 0.9 (0.6)
			Men 60 (14)			Men 0.8 (0.6)	Men 0.4 (0.4)
Humphreys [40]	UK	1995	55 (43, 66)	66	20	Median (IQR) 0.9 (0.4, 1.5)	–
Jäntti [33]	FIN	121	–	–	20	–	–
Kapetanovic [53]	SW	183	52 (12)	63	20	0.9 (0.6)	1.1 (0.7)
Koevoets [39]	NL	508	54 (13)–55 (14)	86	5	1.4 (0.7)	0.6 (0.6)
Kroot [38]	NL	273	ACPA+ 51.1 (15.1)	+ve 62	6	ACPA+ 0.7 (0.4)	–
			ACPA– 52.4 (14.8)	–ve 73		ACPA– 0.7 (0.4)	
Kuiper [45]	NL	332	Postmenopausal women 66 (43–83)	63	6	–	–
			Premenopausal women 36 (17–53)				
			Older men 63 (43–88)				
			Younger men 41 (23–53)				
Kuuliala [48]	SW	85	52.4 (range 18–78)	64	5	0.8 (0.5, 1.2)	0.9 (0.4, 1.3)
Lindqvist [54]	SW	183	51.4 (12.4)	63	10	Median 0.8 (unreported IQR)	Median 1.1 (unreported IQR)
Ling [41]	UK	2158	Median 55 (43–67)	65	5	Median (IQR) 0.9 (0.3, 1.5)	Median (IQR) 0.9 (0.1, 1.6)
Malm [32]	SW	1387	55 (14.1)	70	15	1.0 (0.6)	–
Manivel [47]	SW	773	–	–	5	Median Anti-CII+ 1.0	Median Anti-CII+ 0.5
						Anti-CII– 1.3	Anti-CII– 0.6
Nair [30]	NL	1034	55 (14.5)	66	avg 6.5	1.2 (0.7)	–
Naseem [42]	UK	843	Median (IQR) 53 (40, 65)	64	5	Median (IQR) 0.8 (0.3, 1.4)	Median (IQR) 0.3 (0.3, 1.5)
Thyberg [55]	SW	251	55 (14)	68	8	0.9 (taken from figure)	0.8 (taken from figure)
Verstappen [36]	NL	112	49 (12.4)	76	avg 7	1.1 (0.7)	0.7 (0.6)
Welsing [56]	NL	378	54.8 (14.8)	64	9	Median (IQR) 0.5 (0.2,1.1)	0.6
Welsing [31]	NL	185	55	64	9	Median 0.5	Median 0.6
Wiles [37]	UK	684	Median (IQR) 55 (42, 68)	67	5	Median (IQR) 0.8 (0.3, 1.5)	Median (IQR) 0.9 (0.3, 1.6)
Wiles [57]	UK	528	53 (41–66)	67	5	–	Median (IQR) 0.9 (0.3, 1.6)
Woolf [58]	UK	88	45	72	5	–	–

ACPA = anti-citrullinated protein antibodies, Anti-CII = anticollagen type II antibodies, FIN = Finland, FR = France, HAQ = Health Assessment Questionnaire, IQR = Interquartile range, MX = Mexico, NL = The Netherlands, NZ = New Zealand, RA = rheumatoid arthritis, SD = standard deviation, SW = Sweden, UK = United Kingdom.

**Table 2:** Summary of predictors

			Studies assessing the association		Studies reporting a significant association			
Category	Baseline predictor	Maximum follow-up length	Number of studies	Number of patients	Number of studies	Number of patients	% of total sample in significant studies	Level of evidence^§^
Demographics	Age at baseline	20	18	10.7k	13	9.8k	91.6%	✓✓
	Female gender	15	21	11.3k	13	9.9k	87.4%	✓✓
Patient reported outcomes	HAQ	20	10	4.0k	9	4.0k	99.0%	✓✓
	Pain VAS	15	6	2.9k	5	2.8k	93.6%	✓✓
Disease activity	DAS28	6	5	1.1k	4	888	79.2%	–
	Swollen joint count	20	5	2.6k	1	684	26.6%	✗
	Tender joint count	15	4	2.5k	2	2.1k	84.5%	✓
	Ritchie Index	6	2	233	2	233	100%	–
	CRP	6	4	510	2	279	54.7%	–
	ESR	20	6	2.2k	2	1.6k	72.3%	✓
Serology	RF	20	11	4.6k	3	546	11.9%	✗✗
	ACPA	20	6	3.8k	1	2.0k	52.9%	–
Other	Erosions	20	5	2.7k	1	191	7.0%	✗✗
	Genetics	20	8	4.0k	1	2.2k	54.6%	–
	Morning stiffness	20	3	424	3	424	100%	–
	BMI	15	1	1.6k	1	1.6k	100%	–
	Immigrant status	15	1	1.4k	1	1.4k	100%	–
	SES	5	2	1.0k	2	1.0k	100%	–
	Reproductive factors	15	2^†^	–^†^	2	–^†^		–
	Other biomarkers	20	5	–^†^	2	–^†^		–

^†^Reproductive factors/other biomarkers included are heterogeneous and therefore analysis populations were not summed.

^§^Key.

✓✓ *= ≥ 85% total participants in studies reporting significant association & ≥2000 total participants studied;*

✓ = ≥ 60% & <85% total participants in studies reporting significant association & ≥2000 total participants studied;

– = ≥40% & <60% or <2000 total participants;

✗ = ≥15% & <40% total participants in studies reporting significant association & ≥2000 total participants studied;

✗✗ = <15% total participants in studies reporting significant association & ≥2000 total participants studied.

ACPA = anti-citrullinated protein antibody, BMI = body mass index, CRP = C-reactive protein, DAS28 = Disease activity Score (28), ESR = erythrocyte sedimentation rate, HAQ = Health Assessment Questionnaire, N = number, RF = rheumatoid factor, SES = socioeconomic status, VAS = visual analogue scale.

## Results

A summary of the included studies (*N* = 37), including demographics, follow-up lengths and baseline and final follow-up HAQ scores is presented in Table 1. The studies are presented in alphabetical order of first author to aid cross-reference between tables. Sample sizes ranged from *n* = 25 [23] to *n* = 3666 [24], and follow-up duration from 5 to 20 years (median (IQR) = 6 (5, 10) years). The median age of the patients ranged from 39.1 [25] to 55.6 years [26] (median = 53 years; 27/37 studies reported median age for the entire cohort). The proportions of women ranged from 62% [27], [28] to 100% [29] (median = 66%; 33/37 studies reported the proportion of women). Table 2 summarises the results for each of the predictors assessed in the review.

### Assessment of baseline predictors

#### Demographics

The majority of studies assessing the association between **age** and long-term HAQ score reported that older age at symptom onset was associated with higher HAQ scores at long-term follow-up (18 studies total, 13 (72%) reported a significant association including 11 multivariable analyses) (Table 3); 10.7k patients were included, of which 9.8k were included in analyses that reported a significant association (91.6%). The largest study (*N* = 3666) assessed the association between age and higher HAQ scores over 15 years. The HAQ scores of men aged between 55 and 74 years were, on average, 0.19 (95% CI: –0.01, 0.39) higher and those of men aged ≥75 years were, on average, 1.81 (95% CI: 1.25, 2.36) higher than those men <55 years of age. Older women also had higher HAQ scores compared to younger women, but to a lesser degree (mean difference (95% CI): <55 years = ref, 55–74 = 0.26 (0.12 to 0.40), ≥75 = 0.51 (0.05, 0.98)) [24].

A majority of studies investigating the association between **gender** and later HAQ scores reported that women had significantly higher long-term HAQ scores than men. In total, 21 studies assessed the association between gender and subsequent HAQ scores. Of these, 11 analyses (9 multivariable) reported that women had significantly higher HAQ scores at long-term follow-up than men; one multivariable analysis reported that men had significantly higher HAQ scores than women; three multivariable analyses reported a significant association but the direction of the association was unclear (i.e., the coefficient was labelled “gender” and the reference category (men/women) was not clearly reported); six analyses (4 multivariable) reported no significant association between gender and future HAQ score (Table 3). In total, 11.3k patients were included, of which 9.9k were included in analyses that reported a significant association (87.4%). The average difference between the HAQ scores of women and men ranged from 0.08 [30] to 0.38 [31], based on studies reporting a significant association between female gender and higher HAQ score from linear regression analysis. A study by Malm et al. including 1.4k patients followed for 15 years reported that women had a two and a half times increased odds of having a HAQ score over 0.75 at the 15th year assessment compared to men (OR 2.53, 95% CI: 1.85, 3.46) [32].

**Table 3:** Baseline demographic predictors of follow-up HAQ score

Study details			Predictor: Age		Predictor: Gender		
Authors	*N*	Analysis method	Associated with HAQ	Effect size^a^	Associated with HAQ	Effect size^a^	Adjusted for
*Multivariable analyses*							
Camacho [24]	3666	Multivariable linear random effects model (<55 years used as the reference category)	✓	Men:55–74 *b* 0.19 (–0.01, 0.39)≥75 *b* 1.81 (1.25, 2.36)	✓	Women vs. men:	Age at final follow-up, year recruited to the study
						*b* 0.24 (0.20, 0.29)	*Further adjustment: baseline disease duration and DMARDs within 6 months of symptom onset.
						Further adjustment*: *b* 0.29 (0.25, 0.34)	
				Women:			
				55–74 *b* 0.26 (0.12, 0.40)			
				≥75 *b* 0.51 (0.05, 0.98)			
Malm [32]	1387	Logistic regression	✓	Age at onset (years):	✓	Women vs. men:	Disease duration
		(HAQ cut-off = 0.75)		OR 1.03 (1.02, 1.04)		OR 2.53 (1.85, 3.46)	
Nair [30]	1034	Linear mixed model	✓	Age at onset (years):	✓	“sex”:	Treatment, SHS, HAQ (t-1), DAS28, BMI, RF
				Cohort 1 *b* 0.00, *p* < 0.01		Cohort 1 *b* 0.08, *p* < 0.01	
				Cohort 2 *b* 0.00, *p* = 0.01		Cohort 2 *b* 0.19, *p* < 0.01	
Combe [51]	813	Logistic regression (HAQ cut-off = 0.3)	✓	“older age”:OR 1.91 (1.32, 1.77)	✓	Women vs. men:OR 1.60 (1.02, 2.50)	Baseline: HAQ, pain
Wiles [37]	684	Generalised estimating equations analysis (HAQ cut-off = 1)	✓	<47 years at onset (ref cat):	✓	Women vs. men:	Duration from symptom onset to baseline, Time-varying: morning stiffness, RF, rheumatoid nodules, number of deformed joints
				47–63 OR 1.45 (1.06, 2.00)		OR 1.70 (1.29, 2.24)	
				≥64 OR 3.21 (2.33, 4.42)			
Ahlmen [4]	549	ANCOVA	–	–	✓	Mean (SD) HAQ:	Age
						Men 0.51 (0.56)	
						Women 0.73 (0.68)*p* < 0.01	
Wiles [57]	528	Logistic regression	✓	<47 years (ref cat):	–	–	Year 1 HAQ, Nodules, Knee involvement factor, Tenderness factor (factors created using principal component analysis)
		Model 1 HAQ cut-off = 1,		47–63:			
		Model 2 HAQ cut-off = 1.5		Model 1 OR 2.06 (1.11, 3.83)			
				Model 2 OR 1.62 (0.79, 3.32)			
				≥ 64:			
				Model 1 OR 3.46 (1.77, 6.76)			
				Model 2 OR 2.70 (1.29, 5.67)			
Welsing [56]	378	General linear mixed model	✓	Age at onset (years): *b* 0.01 (0.01, 0.20)	✓	Women vs. men: *b* 0.22 (0.08, 0.36)	Baseline: RF; time-varying: SHS, squared SHS, DAS28
Kroot [38]	273	Multiple regression	✓	Age at entry (years): *b* 0.01, *p* < 0.01	✓	Female gender: *b* –0.128, *p* < 0.05	RF, DAS28, HLA-DR4 gene, ACPA
Hallert [35]	251	Generalised estimating equations analysis	✗	NS – coefficients and confidence interval not reported	✗	NS – coefficients and confidence interval not reported	DMARD use, biologic use, grip force, SOFI-hand, SOFI-upper extremity, SOFI-lower extremity, GAT, pain, walking time
Bjork [49]	189	Projections to latent structure discriminant analysis (HAQ cut-off = 0.08)	✗	Baseline age: VIP 0.22 (“not important”)	✓	“sex”: VIP 1.39 (“important”)	Baseline: HAQ, grip force, SOFI-lower limb, gender, walking speed, GAT, wellbeing, CRP, SOFI-hand, ESR, tender joints, PGA, pain, SOFI-upper limb, swollen joints
Welsing [31]	185	Mixed model (HAQ was log transformed)	✓	Age at onset per year: *b* 0.02, *p* < 0.01	✓	Women vs. men: *b* 0.38 *p* = 0.02	DAS28, Modified SHS, Modified SHS squared, age*modified SHS
Lindqvist [54]	183	Stepwise logistic regression (HAQ cut-off = 1.0)	✗	NS – coefficients and confidence interval not reported	✗	NS – coefficients and confidence interval not reported	Genotype, RF, HAQ, ESR, active joint count
Kapetanovic [53]	183	Hierarchical linear regression	–	–	✗	HAQ at final follow-up:	CCI, DAS, joint damage
						“sex” *b* –0.095, *p* = NS	
						HAQ over time (AUC):	
						“sex” *b* –0.20, *p* < 0.01	
Verstappen [36]	112	Logistic regression (HAQ cut-off = 1)	✓	Age at onset: OR 1.05 (1.01, 1.09)	✗	Women vs. men: OR 0.90 (0.37, 2.17)	Disease duration (natural log transformed)
Contreras-Yanez [25]	107	Multivariable linear regression	✓	Age at baseline (years): *b* 0.10, p = 0.001	✗	NS – coefficients and confidence intervals not reported	Variables tested in univariable analysis: age, gender, disease duration, DAS28, persistence of DMARDs, comorbidity
Kuuliala [48]	85	Logistic regression (HAQ cut-off = 0.9)	✗	Age at entry (years): OR 1.02 (0.97, 1.07)	✓	Women vs. men: OR 5.51 (1.81, 16.8)	RF, Shared epitope, tertiles of soluble E-selectin
Eberhardt [28]	63	Logistic regression (HAQ cut-off = 1)	–	–	✓	Women vs. men: OR 1.02 *p* < 0.01	“[demographic,] clinical, radiographic and laboratory data”
*Univariable analyses*							
Koevoets [39]	508	Generalised estimating equations analysis	–	–	✓	Women vs. men: *b* 0.14 (0.05, 0.24)	–
Kuiper [45]	332	Student’s *t* test	✓	Older men had higher HAQ scores than younger men (*p* < 0.01)	✓	Women had higher HAQ scores than men (*p* < 0.05)	–
Combe [34]	191	Spearman’s test	✗	NS – coefficients and confidence interval not reported	✗	NS – coefficients and confidence interval not reported	–
Jäntti [33]	121	Somers’ *d*	✓	Age at entry:	✗	“sex”:	–
				Somers’ *d* 0.30 (0.16, 0.45)		Somers’ *d* 0.01(–0.31, 0.33)	

See Table 2 for acronym definitions: ACPA, BMI, DAS28, ESR, HAQ, N, RF.

a Brackets indicate 95% confidence interval unless otherwise stated; ANCOVA = analysis of covariance, AUC = area under the curve, *b* = regression coefficient, BL = baseline, CCI = Charlson Comorbidity Index, DMARDs = disease modifying anti-rheumatic drugs, FU = follow-up, GAT = Grip Ability Test, NS = non-significant, OR = odds ratio, PGA = patient global assessment, RA = rheumatoid arthritis, SD = standard deviation, SE = standard error, SHS = Sharp score, SOFI = Signals of Functional Impairment, VIP = variable influence on projection.

#### Patient reported outcomes

Nine (7 multivariable) of the 10 studies that investigated the relationship reported a positive association between higher **disability** at baseline and higher disability at long-term follow-up, whilst the remaining multivariable analysis approached significance (Table 4). Nine of these reported a positive association between baseline HAQ and follow-up HAQ, whilst one used an alternative measure of baseline functional disability [33]. In total, 4.0k patients were included, of which 3.97k were included in analyses that reported a significant association (99.0%). One study (*N* = 191) reported that each unit increase in HAQ score at baseline was associated with a 0.39 (*p* = 0.0001) increase in HAQ score at five years [34]. Another study (*N* = 1.4k) reported that each unit increase in baseline HAQ was associated with a 3.57 (95% CI: 2.84, 4.49) times increased odds of having HAQ > 0.75 at 15th year assessment [32].

**Table 4:** Baseline patient reported outcomes as predictors of follow-up HAQ score

Study details			Predictor: Baseline HAQ		Predictor: Baseline pain		
Study	*N*	Analysis method	Associated with HAQ	Effect size^a^	Associated with HAQ	Effect size^a^	Adjusted for
*Multivariable analyses*							
Malm [32]	1387	Logistic regression(HAQ cut-off = 0.75)	✓	Per unit baseline HAQ:	✓ (VAS)	Per unit baseline pain VAS:	Age, gender, disease duration
				OR 3.57 (2.84, 4.49)		OR 1.02 (1.02, 1.03)	
Bansback [44]	985	Logistic regression(HAQ cut-off = 1.5)	✓	Per unit baseline HAQ:	–	–	Baseline: Carstairs deprivation index, functional grade, haemoglobin level, Larsen score; year 1: functional grade, HAQ, DAS28
				OR 1.70, *p* < 0.01			
Combe [51]	813	Logistic regression(HAQ cut-off = 0.3)	✓	Baseline HAQ cut-off = 0.88:OR 2.90 (2.00, 4.19)	✓ (VAS)	Baseline pain VAS cut-off = 34:	Age, gender
						OR 1.69 (1.17, 2.44)	
Hallert [35]	251	Generalised estimating equations analysis	–	–	✓ (VAS)	Per unit baseline pain VAS:	DMARD use, biologic use, grip force, SOFI-hand, SOFI-upper extremity, SOFI-lower extremity, GAT, pain, walking time
						*b* 0.01, *p* < 0.01	
Combe [34]	191	Spearman correlation & linear regression	✓	ρ 0.47, *p* < 0.01	✓ (VAS)	*ρ* 0.32, *p* < 0.01	ESR, CRP, Ritchie index
				*b* 0.39, *p* < 0.01		*b not reported*	
Bjork [49]	189	Projections to latent structure discriminant analysis (HAQ cut-off = 0.08)	✓	Baseline HAQ cut-off=0.08:	✗ (VAS)	VIP = 0.30 (“not important”)	Baseline: age, gender, grip force, SOFI-lower limb, walking speed, GAT, wellbeing, CRP, SOFI-hand, ESR, tender joints, PGA, SOFI-upper limb, swollen joints
				VIP = 1.97 (“important”)			
Verstappen [36]	112	Logistic regression (HAQ cut-off = 1)	✓	Per unit baseline HAQ:	✓ (VAS)	Per unit baseline pain VAS:	Disease duration (natural log transformed)
				OR 2.63 (1.30, 5.32)		OR 1.02 (0.99, 1.03)	
Eberhardt [28]	63	Logistic regression (HAQ cut-off = 1.0)	✓	Baseline HAQ cut-off = 1.00:	–	–	“[demographic,] clinical, radiographic and laboratory data”
				OR 2.08, *p* < 0.01			
Benton [27]	42	Logistic regression (HAQ cut-off = 0.25) OR are the odds of being in the low HAQ group	✗	Per unit baseline HAQ: OR 0.16 (0.02, 1.01)	–	–	Baseline: DAS, Ritchie index, CRP, Sharp score; one year: DAS, Ritchie, CRP, HAQ
*Univariable analyses*							
Jäntti [33]	121	Somers’ *d*	✓	Somers’ *d* = 0.28 (0.11, 0.45)	–	–	–
Contreras-Yanez [25]	107	Student’s *t* test Comparison groups: HAQ ≤ 0.2 at 5 years, yes/no.	✓	Median (IQR) baseline HAQ:	–	–	–
				HAQ ≤ 0.2 1.4 (0.8–2)			
				HAQ > 0.2 2.1 (1.6–3) *p* < 0.01			

See Table 2 for acronym definitions: DAS28, ESR, HAQ, N.

See Table 3 for acronym definitions: b, DMARD, GAT, OR, PGA, SOFI, VIP.

a Brackets indicate 95% confidence interval unless otherwise stated; CRP = C-reactive protein, IQR = interquartile range, VAS = visual analogue scale, *ρ* = Spearman’s rho.

Four out of six analyses (all multivariable) assessing the relationship reported a positive association between baseline **pain** visual analogue scale (VAS) scores and follow-up HAQ scores; another study approached significance (Table 4). In total, 2.9k patients were included, of which 2.8k were included in analyses that reported a significant association (93.6%). One study using generalised estimating equations analysis over eight years of follow-up, reported that each centimetre increase in pain VAS at baseline was associated with an average increase of 0.06 HAQ score over follow-up [35]. Two studies reported a 2% increased odds in being in a higher HAQ category at follow-up per millimetre increase in pain VAS at baseline, one after seven years of follow-up [36], the other after 15 [32].

#### Disease activity

One multivariable analysis reported a significant positive association between baseline **swollen joint count** and follow-up HAQ scores [37], whilst four other analyses (two multivariable) reported no association. In total, 2.6k patients were included, of which 684 were included in analyses that reported significant results (26.6%).

Two multivariable analyses reported a significant positive association between baseline **tender joint count** and follow-up HAQ score, whilst two other multivariable analyses did not report a significant association (Table 5). In total, 2.5k patients were included, of which 2.0k were included in analyses that reported significant results (84.5%).

**Table 5:** Baseline disease activity measures as predictors of follow-up HAQ score

Study Details			Predictor: Baseline DAS28		Predictor: Baseline joint counts		
Study	*N*	Analysis method	Associated with HAQ	Effect size^a^	Associated with HAQ	Effect size^a^	Adjusted for
*Multivariable analyses*							
Malm [32]	1387	Logistic regression (HAQ cut-off = 0.75)	–	–	✗ (SJC28)	Per baseline swollen joint:	Age, gender, disease duration
						OR 1.02 (0.99, 1.04)	
					✓ (TJC28)	Per baseline tender joint:	
						OR 1.05 (1.03, 1.07)	
Wiles [37]	684	Generalised estimating equations analysis (HAQ cut-off = 1)	–	–	✓ (swelling on different joint sites)	MCP OR 1.57 (1.38, 1.77)	Age at symptom onset, gender, delay to presentation, morning stiffness, RF, number of deformed joints, nodules
		Joint areas clustered using principal component factor analysis				Wrist OR 1.49 (1.34, 1.67)	
					✓ (tenderness factor)	Elbow OR 1.20 (1.09, 1.33)	
						Shoulder OR 1.00 (0.88, 1.14)	
						Knee OR 1.46 (1.33, 1.61)	
						Ankle OR 1.38 (1.24, 1.53)	
						MTP OR 1.15 (1.00, 1.31)	
						Tenderness OR 1.53 (1.37, 1.70)	
Kroot [38]	273	Multiple linear regression	✓	Per unit baseline DAS28: *b* 0.10, *p* < 0.01	–	–	RF, HLA-DR4 gene, ACPA, age, gender
Combe [34]	191	Spearman correlation & linear regression	✓ (univariable)	ρ 0.263	✗ (SJC, univariable)	Baseline swollen joints:	Baseline: ESR, CRP, HAQ, pain VAS
			✗ (multivariable)	*b not reported, not associated*	✓ (TJC, univariable)	ρ 0.00, *p* = 0.45	
					✗ (TJC, multivariable)	Baseline tender joints:	
					✓ (Ritchie, multivariable)	*ρ* 0.27, *p* < 0.01	
						Baseline Ritchie Index:	
						*ρ* 0.29, *p* < 0.01	
						*b* 0.02, *p* = 0.05	
Bjork [49]	189	Projections to latent structure discriminant analysis (HAQ cut-off = 0.08)	–	–	✗ (SJC)	Baseline SJC:	Baseline: age, gender, HAQ, grip force, SOFI-lower limb, walking speed, GAT, wellbeing, CRP, SOFI-hand, ESR, PGA, pain, SOFI-upper limb
					✗ (TJC)	VIP 0.09 (“not important”)	
						Baseline TJC: VIP 0.42 (“not important”)	
Lindqvist [54]	183	Stepwise Logistic regression (HAQ cut-off = 1.0)	–	–	✗ (active joint count)	NS – coefficients and confidence interval not reported	Age, gender, genotype, RF, HAQ, ESR
Verstappen [36]	112	Logistic regression (HAQ cut-off = 1.0)	–	–	✗ (Thomson joint score)	Per unit baseline Thomson score: OR 1.003	Disease duration (natural log transformed)
Benton [27]	42	Logistic regression (HAQ cut-off = 0.25) OR are the odds of being in the low HAQ group	✗	Per unit baseline DAS28: OR 0.72 (0.35, 1.49)	✓ (Ritchie index)	Per unit baseline Ritchie index: OR 0.86 (0.74, 1.00)	Baseline: HAQ, CRP, Sharp score; one year: DAS, Ritchie, CRP, HAQ
*Univariable analyses*							
Koevoet [39]	508	Generalised estimating equations analysis	✓	Per unit baseline DAS28: *b* 0.13 (0.08, 0.18)	–	–	–
Jäntti [33]	121	Somers’ d	–	–	✗ (SJC)	*d* = –0.03 (–0.19, 0.14)	–
Contreras-Yanez [25]	107	Student’s T test	✓	Median (IQR) baseline DAS28:	–	–	–
		Comparison groups: HAQ≤0.2 at 5 years, yes/no		HAQ ≤ 0.2: 6.0 (4.9–6.9)			
				HAQ > 0.2: 6.8 (6.0-7.7).			
				*p* = 0.02			

See Table 2 for acronym definitions: ACPA, CRP, DAS28, ESR, HAQ, N, RF, VAS.

See Table 3 for acronym definitions: b, GAT, NS, OR, PGA, SOFI, VIP.

See Table 4 for acronym definitions: IQR, *ρ*.

a Brackets indicate 95% confidence interval unless otherwise stated; MCP = metacarpophalangeal joint, MTP = metatarsophalangeal joint SJC = swollen joint count, TJC = tender joint count.

Furthermore, two small studies (*N* = 191 and 42) reported a positive association between baseline **Ritchie Index** (which includes a measure of joint tenderness) and subsequent HAQ scores [27], [34]. Thus, the evidence regarding the predictive ability of baseline tender joint counts suggests it may be a useful predictor of long-term HAQ scores, whereas baseline swollen joint count is unlikely to be a predictor of long-term disability.

Two studies (one multivariable) reported that higher **C-reactive protein** (CRP) level was associated with higher long-term functional disability, whilst two multivariable analyses reported no significant association. In total, 510 patients were included, of which 279 were included in analyses that reported significant results (54.7%).

Two multivariable analyses reported a significant association between higher baseline **erythrocyte sedimentation rate** (ESR) and higher follow-up HAQ score, although with small effect sizes (HAQ at 15 years >0.75: OR 1.01 per unit increase in ESR at baseline, 95% CI: 1.002, 1.012 [32]; mean increase in HAQ score at 5 years: 0.008 per unit increase baseline ESR, *p* = 0.006 [34]). Four smaller analyses (three multivariable) reported no significant association (Table 6). In total, 2.2k patients were included, of which 1.6k were included in analyses that reported significant results (72.3%). Thus, there is inconsistent evidence about the relationship between higher CRP and long-term functional disability but ESR is likely to be a weak predictor of HAQ score.

**Table 6:** Baseline blood analyses as predictors of follow-up HAQ score

Study details			Predictor: Baseline ESR/CRP		Predictor: Baseline RF/ACPA		
Study	*N*	Analysis method	Associated with HAQ	Effect size^a^	Associated with HAQ	Effect size^a^	Adjusted for
*Multivariable analyses*							
Humphreys [40]	1995	Generalised estimating equations analysis	–	–	✗ (RF)	RF + vs. RF–: *b* –0.03 (–0.12, 0.05)	Age, gender, smoking status, polynomials of disease duration, year of recruitment
					✓ (ACPA)	ACPA+ vs. ACPA–: *b* 0.12 (0.02, 0.21)	
Malm [32]	1387	Logistic regression (HAQ cut-off = 0.75)	✓ (ESR)	Per unit baseline ESR:	–	–	Age, gender, disease duration
				OR 1.01 (1.00, 1.01)			
Nair [30]	1034	Linear mixed model	–	–	✗ (RF)	RF+ vs. RF–:	Age, gender, treatment, Sharp Score (van der Heijde modification), HAQ (t-1), DAS28, BMI
						Cohort 1 *b* 0.00, *p* = 0.99	
						Cohort 2 *b* 0.00, *p* = 0.90	
Burr [50]	640	Logistic regression (HAQ cut-off = 1)	–	–	✗ (ACPA)	Per unit baseline ACPA titre:	Baseline: age, gender, symptom duration, CRP, RF, HAQ, swollen joint count, tender joint count
						OR 1.00 (0.99, 1.01)	
Kroot [38]	273	Multivariable linear regression	–	–	✓ (RF)	RF+ vs. RF–:	Age, gender, DAS28, HLA-DR4 gene,
					✗ (ACPA)	*b* 0.15, *p* < 0.05	
						ACPA+ vs. ACPA–:	
						*b* 0.00, *p* = NS	
Combe [34]	191	Multivariable linear regression	✓ (CRP)✓ (ESR)	Per unit baseline CRP:	–	–	Baseline: DAS, swollen joint count, tender joint count, HAQ, pain VAS
				*b* 0.01 (*p* < 0.01)			
				Per unit baseline ESR:			
				*b* 0.01 (*p* < 0.01)			
Bjork [49]	189	Projections to latent structure discriminant analysis (HAQ cut-off = 0.08)	✗ (CRP)	Per unit baseline CRP:	–	–	Baseline: Age, gender, HAQ, grip force, SOFI-lower limb, walking speed, GAT, wellbeing, swollen joint count, SOFI-hand, tender joint count, PGA, pain, SOFI-upper limb
			✗ (ESR)	VIP 0.63 (“not important”)			
				Per unit baseline ESR:			
				VIP 0.49 (“not important”)			
Welsing [31]	185	General linear mixed model	–	–	✓ (RF)	RF+ vs. RF–:	Baseline: age, sex; time-varying: Sharp score, squared Sharp score, DAS28
						*b* 0.19 (0.03, 0.35)	
Lindqvist [54]	183	Stepwise logistic regression (HAQ cut-off = 1.0)	✗ (ESR)	NS – coefficients and confidence intervals not reported	✗ (RF)	NS – coefficients and confidence intervals not reported	Age, gender, genotype, HAQ, active joint count
Verstappen [36]	112	Logistic regression (HAQ cut-off = 1)	✗ (ESR)	Per unit baseline ESR:	–	–	Disease duration (natural log transformed)
				OR 1.00 (0.99, 1.02)			
Kuuliala [48]	85	Logistic regression (HAQ cut-off = 0.9)	–	–	✗ (RF)	RF+ vs. RF–:	Age, gender, shared epitope, tertiles of soluble E-selectin
						OR 1.09 (0.33, 3.57)	
Benton [27]	42	Logistic regression (HAQ cut-off = 0.25) OR are the odds of being in the low HAQ group	✗ (CRP)	Per unit baseline CRP:	–	–	Baseline: DAS, Ritchie index, HAQ, Sharp score; one year: DAS, Ritchie index, CRP, HAQ
				OR 0.99 (0.96, 1.03)			
*Univariable analyses*							
Koevoets [39]	508	Generalised estimating equations analysis	–	–	✗ (RF)	RF+ vs. RF–:	–
					✗ (ACPA)	*b* –0.03 (–0.13, 0.08)	
						ACPA+ vs. ACPA–:	
						*b* –0.03 (–0.13, 0.07)	
Thyberg [55]	251	Chi-Square (HAQ cut-off=1)	–	–	✗ (ACPA)	NS difference between proportion of ACPA+ patients between HAQ subgroups	–
Jäntti [33]	121	Somers’ d	✗ (ESR)	*d* = 0.12 (–0.04, 0.28)	✗ (RF)	*d* = –0.18 (–0.62, 0.26)	–
Contreras-Yanez [25]	107	Student’s *t* test Comparison groups: HAQ ≤ 0.2 at 5 years, yes/no	–	–	✗ (RF)	Proportion baseline RF+:	–
					✗ (ACPA)	HAQ ≤ 0.2 82.1%	
						HAQ > 0.2 82.6%	
						*p* = 1.00	
						Proportion baseline ACPA+:	
						HAQ ≤ 0.2 85.7%	
						HAQ > 0.2 87.0%	
						p=1.00	
Woolf [58]	88	Calculated sensitivity and specificity of having HAQ > 0 at 5 years	✓ (“raised CRP”)	Specificity/sensitivity:	✓ (RF)	Specificity/sensitivity:	–
				93/74		64/37	
Genevay [23]	25	Mann-Whitney	–	–	✗ (RF)	Mean HAQ:	–
						RF+ 0.78	
						RF– 0.80	
						*p* = NS	

See Table 2 for acronym definitions: ACPA, BMI, CRP, DAS, ESR, HAQ, N, RF, VAS.

See Table 3 for acronym definitions: b,GAT,NS,OR,PGA, SOFI, VIP.

a Brackets indicate 95% confidence interval unless otherwise stated.

Of the five studies which assessed the association, three univariable analyses and one multivariable analysis reported a positive association between baseline **Disease Activity Score (28)** (DAS28) and follow-up HAQ scores, whilst one multivariable analysis did not report a significant association (Table 5). In total, 1.1k patients were included, of which 888 were included in analyses that reported a significant association (79.2%). The average increase in HAQ score at follow-up per unit increase in baseline DAS28 ranged from 0.100 [38] to 0.130 [39], based on analyses reporting significant associations from linear regressions.

#### Serology

**Rheumatoid factor** (RF) positivity did not predict higher HAQ scores in the majority of included studies. Eight analyses (four multivariable) reported no association between RF positivity and later HAQ scores, whilst three analyses (two multivariable) did report an association (Table 6). The two largest multivariable analyses (mean difference in HAQ between RF+ and RF− = –0.03 (95% CI: –0.12, 0.05) [*N* = 1995] [40], 0.00014 (*p* = 0.9959) [*N* = 1034] [30]) and the largest univariable analysis (mean difference in HAQ between RF+ and RF– = –0.027 (95% CI: –0.130, 0.076) [*N* = 508] [39]) found no association. In total, 4.6k patients were included, of which 546 were included in analyses that reported significant results (11.9%).

The largest analysis assessing the association between **anti-citrullinated protein antibodies** (ACPA) positivity and subsequent HAQ scores reported a significant association (*N* = 1995; mean difference in HAQ between ACPA+ and ACPA– = 0.12 (95% CI: 0.02, 0.21)) [40], but five other analyses (two multivariable) found no association (Table 6). In total, 3.8k patients were included, of which 2.0k were included in analyses that reported significant results (52.9%). Thus at present the literature is equivocal as to whether ACPA positivity is a useful predictor of increased long-term functional disability.

#### Erosions

Four out of five studies (three multivariable) reported no significant association between **erosion score** at baseline and subsequent higher HAQ scores (Table 7). One univariable analysis reported a significant correlation [34], but with a low Spearman's rho (*ρ* = 0.167) indicating a weak relationship. Of the 2.7k patients included in analyses assessing the association, only 191 were included in the analysis that reported a significant association (7.0%). Therefore, based on current evidence, baseline erosions are not a predictor of long-term functional disability in patients with early inflammatory arthritis.

**Table 7:** Miscellaneous predictors of long-term functional disability

Predictor	Study	*N*	Analysis method	Associated with HAQ	Effect size^a^	Adjusted for
***BMI***	Ajeganova [26]	1596	Multivariable linear regression	✓ (BMI)	Per unit baseline BMI:	Age, duration of follow-up, gender, ever glucocorticoid use, ever biologic use
					*b* 0.02 (0.01, 0.03)	
***Erosions***	***Multivariable analyses***					
	Malm [32]	1387	Logistic regression (HAQ cut-off = 0.75)	✗ (x-ray erosions)	Baseline erosions yes vs. no:	Age, gender, disease duration
					OR 1.24 (0.93, 1.66)	
	Bansback [44]	985	Logistic regression (HAQ cut-off = 1.5)	✗ (Larsen score)	Per unit baseline Larsen Score:	Carstairs deprivation index; baseline: functional grade, HAQ, haemoglobin level; Year 1: functional grade, HAQ, DAS28
					OR 1.01 (*p* = 0.20)	
	Benton [27]	42	Logistic regression (HAQ cut-off = 0.25)	✗ (Sharp score)	Per unit baseline Sharp Score:	Baseline: DAS, Ritchie index, CRP, HAQ; one year: DAS, Ritchie, CRP, HAQ
			OR are the odds of being in the low HAQ group		OR 0.96 (0.84, 1.08)	
	***Univariable analyses***					
	Combe [34]	191	Spearman correlation	✓ (Sharp score)	ρ 0.17, *p* = 0.04	–
	Jäntti [33]	121	Somers’ *d*	✗ (Larsen score)	*d* = 0.01 (–0.62, 0.26)	–
***Genetic factors***	***Multivariable analyses***					
	Ling [41]	2158	Generalised Linear Latent and Mixed Models	✓ (Amino acids at HLA-DR4)	Valine 11 *b* 0.02 (0.00, 0.04)	Age at symptom onset and disease duration at follow-up
			Comparison between amino acid at a particular position vs. all other amino acids at that position		Proline 11 *b* –0.03 (–0.07, 0.00)	
					Serine 11 *b* –0.02 (–0.04, 0.00)	
					Arginine 71 *b* 0.02 (0.00, 0.04)	
					Alanine 71 *b* –0.06 (–0.09, –0.02)	
					Glutamic acid 71 *b* –0.06 (–0.09, –0.03	
					Other positions not significant	
	Kroot [38]	273	Multiple regression	✗ (HLA-DR4)	HLA-DR4+ vs. HLA-DR4–:	Age, gender, RF, DAS, ACPA
					*b* < 0.001, *p* = NS	
	Lindqvist [54]	183	Stepwise logistic regression (HAQ cut-off = 1.0)	✗ (HLA-DRB alleles)	NS – coefficients and confidence interval not reported	Age, gender, RF, HAQ, ESR, active joint count
	Kuuliala [48]	85	Logistic regression (HAQ cut-off = 0.9)	✗ (shared epitope)	Shared epitope:	Age, gender, sE-selectin, RF
					None OR 1 (ref)	
					single copy OR 0.41 (0.07, 2.35)	
					double copy OR 1.14 (0.21, 6.23)	
	***Univariable analyses***					
	Naseem [42]	843	Mann-Whitney	✗ (PTPN22)	No association between PTPN22 SNPs and HAQ at 5 years	–
	Combe [34]	191	Kruskal-Wallis test	✗ (HLA-DRB1)	NS – coefficients and confidence interval not reported	–
	Jäntti [33]	121	Somers’ *d*	✗ (HLA-B27)	HLA-B27+ vs HLA-B27–:	–
					*d* = –0.01 (–0.33, 0.31)	
	Eberhardt [52]	99	Wilcoxon/Mann-Whitney *U* test	✗ (HLA-DRB1/DQB antigens)	NS difference in HAQ	–
***Immigrant status***	Andersson [43]	1430	Mann-Whitney *U*	✓ (Immigrant)	Mean HAQ at 5 years:	–
					Immigrants 0.69	
					Non-immigrants 0.56	
					*p* = 0.04	
***Morning stiffness***	Verstappen [36]	112	Logistic regression (HAQ cut-off = 1)	✓ (morning stiffness)	Per minute baseline morning stiffness:	Disease duration (natural log transformed)
					OR 1.01 (1.00, 1.02)	
	Combe [34]	191	Spearman correlation	✓ (morning stiffness)	*ρ* 0.21, *p* = 0.05	–
	Jäntti [33]	121	Somers’ D	✓ (morning stiffness)	*d* = 0.28 (0.00, 0.55)	–
***Other Biomarkers***	***Multivariable analyses***					
	Humphreys [40]	1995	Generalised estimating equations analysis	✓ (anti-CarP)	Anti-CarP+ vs. anti-CarP–:	Age, gender, smoking status, polynomials of disease duration, year of recruitment
					*b* 0.12 (0.02, 0.21)	
	Kuuliala [48]	85	Logistic regression (HAQ cut-off = 0.9)	✓ (sE-selectin)	Level of sE-selectin:	Age, gender, shared epitope, RF
					1st tertile OR 1 (ref)	
					2nd tertile OR 2.45 (0.70, 8.59)	
					3rd tertile OR 4.18 (1.15, 15.22)	
	***Univariable analyses***					
	Manivel [47]	773	ANOVA	✗ (Anti-CII)	NS difference between Anti-CII+ and Anti-CII- patients	–
	Forslind [46]	92	Mann-Whitney U	✗ (AFA)	NS difference between AFA+ and AFA− patients	–
	Genevay [23]	25	Mann-Whitney U	✗ (APF)	Mean HAQ at follow-up:	–
				✗ (AKA)	APF+ 0.94	
					APF– 0.75	
					*p* = NS	
					AKA+ 0.82	
					AKA– 0.78	
					*p* = NS	
***Reproductive factors***	Camacho [29]	1872	Linear random effects model	✓ (parous)	Parous vs. nulliparous at baseline:	Age, disease duration, SES, smoking RF, ACPA, comorbidities, ACR RA criteria
					*b* –0.19 (–0.34, –0.05)	
	Kuiper [45]	332	Student’s t test	✓ (menopause)	Postmenopausal women had higher HAQ than premenopausal women (*p* < 0.01)	–
***Socioeconomic status***	Bansback [44]	985	Logistic regression (HAQ cut-off = 1.5)	✓ (Carstairs deprivation index)	Carstairs index:	Baseline: HAQ, functional grade, haemoglobin level, Larsen score; year 1: functional grade, HAQ, DAS28
					1 OR 1.0 (ref)	
					2 OR 0.78 (*p* = 0.44)	
					3 OR 1.44 (*p* = 0.24)	
					4 OR 1.73 (*p* = 0.08)	
					5 OR1.98 (*p* = 0.04)	
	Eberhardt [28]	63	Logistic regression (HAQ cut-off = 1.0); education groups (Years): 0–9, 10–11, ≥12	✓ (Years of education)	Per education group change:	“[demographic,] clinical, radiographic and laboratory data”
					OR 0.87 (*p* = 0.05)	

See Table 2 for acronym definitions: ACPA, BMI, CRP, DAS28, ESR, HAQ, RF, SES.

See Table 3 for acronym definitions: *b*, NS, OR, RA.

See Table 4 for acronym definitions: *ρ*.

a Brackets indicate 95% confidence interval unless otherwise stated; ACR = American College of Rheumatology, AFA = antifilaggrin antibodies, AKA = antikeratin antibody, ANOVA = analysis of variance, Anti-CarP= anticarbamylated protein antibodies, Anti-CII = anticollagen type II antibodies, APF = antiperinuclear factor, sE-selectin = soluble E-selectin, SNP = single nucleotide polymorphism.

#### Morning stiffness

All three analyses (one multivariable; total patients included = 424) that assessed the relationship between **morning stiffness** and long-term HAQ score reported a positive association (Table 7). One study reported a low Spearman's rho (*ρ* = 0.211) indicating a weak relationship [34], another study reported a 1% increased odds of having a HAQ > 1 after seven years per minute increase in morning stiffness at baseline compared to no morning stiffness (max = 180; OR 1.008, 95% CI: 1.001, 1.016) [36]. This suggests that baseline morning stiffness may be weakly associated with long-term functional disability, but all the studies reporting on this relationship were relatively small (*N* < 200).

#### Genetic factors

Eight analyses (four multivariable) assessed the association between **RA susceptibility genes** (HLA and PTPN22 variants) and long-term functional disability (Table 7). One large study reported significant associations between different amino acids at positions 11, 71 and 74 of HLA-DRB1 and small increases or decreases in disability over five years [41]. Of the other studies, seven studies examined different HLA regions as the independent variable and one study examined PTPN22 variants [42], all reporting no significant associations. Therefore, the published literature suggests that specific amino acids at different positions of the HLA-DRB1 gene are weakly associated with long-term disability. Other genes within the HLA region do not predict long-term functional disability, with little research on other genetic regions.

#### Other factors

The one multivariable analysis (*N* = 1.6k) which assessed **body mass index** (BMI) as a predictor of long-term HAQ reported that each unit increase in BMI at baseline was associated with a 0.2 increase in HAQ at 15 years follow-up (Table 7) [26].

The one univariable analysis (*N* = 1.4k) which assessed whether **immigrant status** predicted long-term HAQ score reported that immigrants to Sweden had significantly higher HAQ scores after 15 years compared to non-immigrants (Table 7) [43]. Bansback et al. reported that those in the highest category (i.e., most deprived) of the Carstairs Deprivation Index, a measure of **socioeconomic status**, at baseline had an almost two-fold increased adjusted odds of having a HAQ > 1.5 after five years, compared to those in the lowest category (OR 1.984, *p* = 0.044, *N* = 985) [44]. Eberhardt et al. (*N* = 63) reported that compared to those with 0–9 years of education, patients who had 10–11 years had a 13% lower odds of having HAQ score>1.0 at five years and those with ≥12 years of education had 26% lower odds, after adjusting for confounders [28].

Two studies reported on **reproductive factors**. One reported a significant association between being parous at baseline vs. nulliparous and subsequent lower HAQ score over 15 years of follow-up (*N* = 1.9k) [29], and the other reported that women who were postmenopausal at baseline had significantly higher HAQ scores six years later than women who were premenopausal (*N* = 332) [45]. However, the latter study did not control for age.

Four studies examined the association between **other biomarkers** and subsequent HAQ scores (Table 7). No association was found between antifilaggrin antibody, antiperinuclear factor, antikeratin or anticollagen type II antibody status and subsequent HAQ scores [23], [46], [47]. However, anti-carbamylated protein antibody positivity and being in the highest tertile of sE-selectin level were associated with higher long-term HAQ score [40], [48].

## Discussion

This systematic review identified 37 studies that assessed the association between a total of 20 baseline variables and subsequent long-term functional disability, as measured by the HAQ, in patients with inflammatory arthritis. There was highly consistent evidence of an association between female gender, higher baseline age and higher baseline HAQ score, with subsequent higher HAQ scores. There was moderately consistent evidence of an association between higher baseline pain, DAS28 and morning stiffness and subsequent increased HAQ score. However in general, studies reported weak or no association between higher baseline swollen joint count, erosions, HLA genetic variations or RF positivity with later HAQ scores. The literature is equivocal regarding the relationship between ACPA positivity and subsequent HAQ scores.

The findings of this review are in agreement with a review carried out by Scott et al. in 2003, which reported that women and those of older age at baseline were more likely to have high disability in the future [16]. Scott et al. also found that higher pain at baseline was associated with higher subsequent disability. However, Scott et al. reported that RF positivity and a high number of erosions were associated with increased disability at follow-up. The association between more erosions and subsequent higher HAQ score was also reported in a review by Bombardier et al. [59]. This is likely to be because both of these previous reviews included patients with any disease duration, whilst the current review only included studies confined to early arthritis patients (symptom duration ≤2 years at baseline) who may not yet have developed erosions.

Baseline HAQ score was the only variable that was shown to be associated with higher HAQ score at follow-up consistently across all studies assessing the relationship (with nine studies reporting a significant association and one study trending towards significance). Higher levels of pain and morning stiffness at baseline may also be useful predictors of subsequent higher HAQ score, although the evidence for this is weaker. Furthermore, four out of five studies assessing the relationship reported a significant relationship between baseline DAS28 and later functional disability. However, the longest follow-up of these studies was six years.

Also of clinical interest are the results of studies assessing the association between RF and ACPA positivity and later HAQ scores. None of the three large cohort studies with over 1000 patients at baseline reported a significant association between RF positivity and later higher HAQ scores and only one study out of six reported a significant association between ACPA positivity at baseline and later higher HAQ scores. However, this was by far the largest study to assess the association, including almost 2000 patients in the analysis [40].

This review has a number of strengths. Limiting the review to studies of patients with early arthritis allows us to examine which factors early in the disease process predict later functional disability. Furthermore, we have stratified the presentation of results into multivariable and univariable analyses, and then sorted within these sets based on the sample size of the studies. Therefore analyses with high power which control for confounding are presented first, allowing the reader to easily assess the quality of the studies presented.

A drawback to this review is that a meta-analysis could not be performed due to the heterogeneity between the studies. Almost every study assessed the association between baseline variables and subsequent HAQ scores in a different way, using different analysis techniques and controlling for different combinations of covariates. Any meta-analysis combining these studies would be uninterpretable. Furthermore, we have included all studies published since 1990 that met the inclusion criteria. Thus secular trends in disease severity could be influencing the results of the review [60], [61] or differences in the available treatments and treatment strategies over time may mean that studies published over this period are not comparable.

The majority of studies included within the review were judged to be of moderate quality (Supplementary File 2). Studies often did not report on the amount of missing data. Other studies used complete case analyses, which could mean that the results of the studies are biased. Furthermore, studies often included covariates in analyses but only reported on the primary predictor defined in the research question. Therefore, these covariates could not be included in the review, despite contributing to the analyses.

In conclusion, this review has demonstrated that female gender and higher baseline age, HAQ score, pain score and duration of morning stiffness have been consistently reported to predict long-term increased functional disability. Furthermore, most studies assessing the association reported no association between RF and erosion and early IA patients’ long-term disability. This study indicates the relative importance of patient reported outcomes over blood test results in predicting the long-term prognosis (in terms of physical disability) of patients with IA.
