# Supplementary material for: Baseline patient reported outcomes are more consistent predictors of long-term functional disability than laboratory, imaging or joint count data in patients with early inflammatory arthritis: A systematic review
Source: Semin Arthritis Rheum. 2018 Dec;48(3):384–98. doi: 10.1016/j.semarthrit.2018.03.004 (PMC6562164; doi:10.1016/j.semarthrit.2018.03.004)
Supplement: Supplementary file 1 — Supplementary material [file mmc1.docx]

***Supplementary file 1 - Search Strategy***

The search strategy below is split into five sections which each assess a particular part of the research question. Terms 1-4 select the correct patient group. Terms 6-11 select the correct outcome. Terms 13-19 select the correct study design. Terms 21-36 select studies that assess the association between variables and outcome. Term 38 combines these four sections. Terms 39-58 are each an exclusion criterion and term 59 removes studies from those included by term 38 that have exclusion criteria in them.

1. exp Arthritis, Rheumatoid/
2. RA.mp.
3. (Undifferentiated adj3 arthritis).mp.
4. Inflammatory $arthritis.mp.
5. or 1-4
6. health assessment questionnaire.mp.
7. HAQ.mp.
8. MHAQ.mp.
9. HAQ-DI.mp.
10. functional disability.mp.
11. functional outcome.mp.
12. or 6-11
13. exp Observational Study/
14. exp Longitudinal Studies/
15. follow$ up$.mp.
16. followup$.mp.
17. exp Cohort Studies/
18. exp Prospective Studies/
19. long term.mp.
20. or 13-19
21. associat$ adj25 ( health assessment questionnaire OR HAQ OR functional outcome OR functional disability OR baseline measure$ OR activity limitation).mp.
22. predict$ adj25 ( health assessment questionnaire OR HAQ OR functional outcome OR functional disability OR baseline measure$ OR activity limitation).mp.
23. predictor of outcome.mp.
24. predictive factor$
25. baseline predictor
26. correlat$ adj25 ( health assessment questionnaire OR HAQ OR functional outcome OR functional disability OR baseline measure$ OR activity limitation).mp.
27. explained by adj25 ( health assessment questionnaire OR HAQ OR functional outcome OR functional disability OR baseline measure$ OR activity limitation).mp.
28. relation$ adj25 ( health assessment questionnaire OR HAQ OR functional outcome OR functional disability OR baseline measure$ OR activity limitation).mp.
29. significantly adj25 ( health assessment questionnaire OR HAQ OR functional outcome OR functional disability OR baseline measure$ OR activity limitation).mp.
30. determinant adj25 ( health assessment questionnaire OR HAQ OR functional outcome OR functional disability OR baseline measure$ OR activity limitation).mp.
31. influence$ adj25 ( health assessment questionnaire OR HAQ OR functional outcome OR functional disability OR baseline measure$ OR activity limitation).mp.
32. variation$ adj25 ( health assessment questionnaire OR HAQ OR functional outcome OR functional disability OR baseline measure$ OR activity limitation).mp.
33. effect$ adj25 ( health assessment questionnaire OR HAQ OR functional outcome OR functional disability OR baseline measure$ OR activity limitation).mp.
34. related adj25 ( health assessment questionnaire OR HAQ OR functional outcome OR functional disability OR baseline measure$ OR activity limitation).mp.
35. impact adj25 ( health assessment questionnaire OR HAQ OR functional outcome OR functional disability OR baseline measure$ OR activity limitation).mp.
36. prognostic
37. or 21-36
38. 5 AND 12 AND 20 AND 37
39. randomised control$ trial.mp.
40. randomized control$ trial.mp.
41. RCT.mp.
42. clinical trial.mp
43. cross sectional$.mp.
44. case report$.mp.
45. case stud$.mp.
46. child$.mp.
47. review.mp.
48. juvenile.mp.
49. adolescent.mp.
50. infant.mp.
51. Animals/
52. canine.mp.
53. exp Dogs/
54. feline.mp
55. exp Cats/
56. exp Mice/
57. exp Rats/
58. or 39-58
59. 38 NOT 58

**Supplementary File 2 – Quality Assessment**

The studies included in the review were assessed in terms of the quality of the study design and reporting based on six criteria, based on a previously developed framework by Pasma et al [1]:

1. Whether the sampling frame and the age and sex of the included participants was clearly reported
2. Whether the baseline characteristics of patients who refused to take part in the study were compared with the baseline characteristics of patients who were included
3. Whether the number of patients who attended final follow-up was clearly reported
4. Whether the patients were recruited using consecutive or stratified sampling
5. Whether missing data was low and therefore would not have affected results (<5% missing) or whether missing data were dealt with appropriately
6. Whether any conflicts of interest were clearly reported

If a study did not clearly report on each of these items, then the study scored a 0 for that item. If a study reported clearly on an item then they scored a 1. The proportion of studies scoring 1 will be reported for each critieria and a total score will be calculated for each study, based on summation of the criterions. If a paper pointed the reader to an early publication that detailed the methods of the study, this earlier study was also screened.

**Results**

The table below shows the proportion of papers within the review that reported well on each criterion and the proportion who did not report sufficiently. For criteria 1, 3 and 4 there was good a good level of reporting across studies. However no studies attempted to compare the patients who refused to take part in the analysis compared to patients who were included. Of course this can be difficult, as by definition patients have refused to take part in the study and therefore there will be little data on these patients. However at least a number of patients who were approached by the research team and who refused could be reported, which would give some idea about the level of selection bias within the study.

*Table – proportions of included papers with sufficient and insufficient information on each of the criterions in the quality assessment*

| Quality Criterion | N (%) scoring 1 | N (%) scoring 0 |
| --- | --- | --- |
| 1) Sample frame, age and sex clearly reported | 33 (89.2) | 4 (10.8) |
| 2) Comparison of baseline characteristics between those who participated and those who did not | 0 (0) | 37 (100) |
| 3) Number attended final follow-up clearly stated | 28 (75.7) | 9 (24.3) |
| 4) Consecutive or stratified sampling | 31 (83.8) | 6 (16.2) |
| 5) Missing data dealt with appropriately | 5 (13.5) | 32 (86.5) |
| 6) Conflicts of interest reported clearly | 15 (40.5) | 22 (59.5) |

Information on missing data was very rarely reported. It is hard to believe that these studies, all of which have at least five years of follow-up had no missing data on any of the variables used in the analysis. Therefore, with no report as to the amount of missing data, it is hard to judge these papers in terms of how biased they may be due to missing data.

Many papers were published without information on any competing interests of the authors. It is unsafe to assume that if there is no information on competing interests of authors (i.e. there is no section entitled “competing interests” or no statement saying “the authors had no competing interests to declare) then there were no conflicts of interest in the study. Again, as this information was so sparsely reported, it is hard to make an informed judgement about the level of bias that may have been introduced into the studies due to competing interests.

The mean total score was 3.0 (SD 0.9) and the median score was 3 (IQR 3, 4). As is clear from the table, most studies got to three points by reporting well on criterion points 1, 3 and 4.

**Discussion**

The quality assessment procedure used within this systematic review has flagged up that many papers do not report on a number of aspects that are important when trying to judge a paper’s quality. Missing data is a ubiquitous problem in epidemiological research and yet very few papers even give the issue a passing comment, meaning that it is very hard to judge how biased this review was due to unknown levels of missing data. Furthermore disclosure statements were routinely lacking. The papers that included a disclosure statement tended to be the papers published more recently, indicating that the drive towards more transparency in science over the past decade has been effective. Journals now stipulating that all authors need to make disclosure statements before a paper can be published is likely to be driving this.

Therefore, the quality of reporting of the studies included in this review was relatively mediocre , meaning that it is difficult to give any clear judgement about how biased the papers within the review are. It is clear that papers published more recently tended to have more aspects reported clearly; this could be in part due to the publication of the STROBE statement, the first version of which was published in 2004 [2].

REFERENCES

1 Pasma A, van't Spijker A, Hazes JM et al. Factors associated with adherence to pharmaceutical treatment for rheumatoid arthritis patients: a systematic review. *Semin Arthritis Rheum* 2013;**43**:18-28.

2 von Elm E., Altman DG, Egger M et al. The Strengthening the Reporting of Observational Studies in Epidemiology (STROBE) statement: guidelines for reporting observational studies. *J Clin Epidemiol* 2008;**61**:344-49.

| **Study Characteristics**  ***Supplementary file 3 – Data extraction form*** |  |
| --- | --- |
| Citation. |  |
| Year of study. |  |
| Country of origin. |  |
| No. of patients. |  |
| Study follow-up period. |  |
| Part of large prospective cohort? (e.g. NOAR) |  |
| IP or RA? | - IP - 1987 RA criteria - 2010 RA criteria |
| Study design. | - Prospective - Retrospective |
| Patient baseline demographics. | Women, N (%):  Men, N (%):  Age at study baseline: mean / median  Maximum disease duration: mean / median  Mean/median disease duration: |
| Functional disability at baseline. | HAQ:  HAQ-DI:  MHAQ: |
| Functional Disability at final time point. |  |
| Statistical method used | - Univariate   Specify:   - Multivariate   Specify: |
| Baseline covariate list: |  |
| Results of analysis: |  |
